# The Underappreciated Role of Epithelial Mesenchymal Transition in Chronic Obstructive Pulmonary Disease and Its Strong Link to Lung Cancer

**DOI:** 10.3390/biom11091394

**Published:** 2021-09-21

**Authors:** Malik Quasir Mahmood, Shakti D. Shukla, Chris Ward, Eugene Haydn Walters

**Affiliations:** 1 Faculty of Health, School of Medicine, Deakin University, Waurn Ponds, VIC 3216, Australia; malik.mahmood@deakin.edu.au; 2 Graduate School of Pharmacy, University of Technology Sydney, Ultimo, NSW 2007, Australia; shakti.shukla@utas.edu.au; 3 Faculty of Medical Sciences, Translational and Clinical Research Institute, Newcastle University Medical School, Newcastle University, Newcastle upon Tyne NE1 7RU, UK; chris.ward@newcastle.ac.uk; 4 School of Medicine, University of Tasmania, Hobart, TAS 7000, Australia

**Keywords:** epithelial-mesenchymal transition, chronic obstructive pulmonary disease, lung cancer, therapy

## Abstract

The World Health Organisation reported COPD to be the third leading cause of death globally in 2019, and in 2020, the most common cause of cancer death was lung cancer; when these linked conditions are added together they come near the top of the leading causes of mortality. The cell-biological program termed epithelial-to-mesenchymal transition (EMT) plays an important role in organ development, fibrosis and cancer progression. Over the past decade there has emerged a substantial literature that also links EMT specifically to the pathophysiology of chronic obstructive pulmonary disease (COPD) as primarily an airway fibrosis disease; COPD is a recognised strong independent risk factor for the development of lung cancer, over and above the risks associated with smoking. In this review, our primary focus is to highlight these linkages and alert both the COPD and lung cancer fields to these complex interactions. We emphasise the need for inter-disciplinary attention and research focused on the likely crucial roles of EMT (and potential for its inhibition) with recognition of its strategic place mechanistically in both COPD and lung cancer. As part of this we discuss the future potential directions for novel therapeutic opportunities, including evidence-based strategic repurposing of currently used familiar/approved medications.

## 1. Introduction

Epithelial-mesenchymal transition (EMT) refers to a biological process in which basal cells in a polarised epithelium with cell-cell contacts and attached to the basal membrane differentiate into (myo) fibroblast-type mesenchymal cells [1]. This process demonstrates the plasticity of epithelial cells that can also undergo the reverse process, i.e., the mesenchymal-epithelial transition (MET) [2,3]. EMT involves the loss of epithelial polarity due to the disassembly of cell-cell contacts such as adherent junctions (E-cadherin) or tight junctions (zonula occludens-1) and, on the other hand, gain of expression of mesenchymal proteins such as α-smooth muscle actin [4], vimentin and/or fibronectin [5,6]. Enhanced production of extracellular matrix (ECM) components (fibronectin and collagens) and tissue matrix-degrading enzymes, matrix metalloproteinases (MMP2 and MMP9) gives the ability to lay down new and altered matrix which also leads to the loss of adherence to the ECM and gives the cells a migratory capacity.

Historically, epithelial and mesenchymal cells have been differentiated histologically on the basis of their characteristic visual appearances and the morphology of the organised multicellular structures they create [7]. A typical epithelium is a sheet of cells, often one cell thick (or pseudo-stratified), with individual epithelial cells all attached to the basement membrane, and abutting each other in a uniform array. Regularly spaced cell-cell junctions with adhesion proteins between neighbouring epithelial cells hold them tightly together and inhibit the movement of individual cells away from the epithelial monolayer. Mutual adhesiveness allows an epithelial sheet to enclose a three-dimensional space and provides it with structural definition and mechanical integrity. The epithelial sheet itself is polarized, meaning that the apical and basal surfaces are likely to be visually different, adhere to different substrates and have different functions [8].

Mesenchymal cells, on the other hand, generally exhibit neither regimented structure nor tight intracellular adhesion, although they typically have intense functional interaction with molecules in surrounding matrix. Mesenchymal cells form structures that are irregular in shape and not uniform in composition or density. Adhesions between mesenchymal cells are less strong than for their epithelial counterparts, allowing for increased migratory capacity [9]. Mesenchymal cells also have a more extended and elongated shape, relative to epithelial cells, though they possess front-to-back leading-edge polarity. Moreover, mesenchymal migration is mechanistically different from epithelial movement; the latter move as a sheet en-lock, whereas for the former migration is considerably more dynamic. Mesenchymal cells move individually. Elizabeth Hay, who first described EMT, illustrated the fundamental differences in such movement between embryogenesis (subtle/controlled) and tumorigenesis (aggressive/uncontrolled) to define these distinct EMT mechanisms [1]. Such differences have led to division of EMT into three general types.

## 2. Types of EMT

**Type I EMT** is that involved in tissue and organ formation during embryogenesis [10], and is implicated in the differentiation of the mesoderm from the epithelium and in the generation of neural crest cells. In this way, the primary mesenchyme is formed from the epiblast (primitive epithelium) [11,12,13,14].

**Type II EMT** is involved in non-neoplastic fibroblast recruitment in chronic inflammation-related wound healing, tissue repair and tissue regeneration/remodelling [15,16,17,18,19]. However, with uncoordinated and uncontrolled tissue repair processes, EMT is associated with tissue fibrosis; accumulated fibroblasts secrete collagen fibres, leading to organ dysfunction.

**Type III EMT** refers to the acquisition of a migratory phenotype by malignant epithelial cells and is associated with tumour invasiveness and metastasis [20,21]. Epithelial cells lose their polarity and detach from the basal membrane, migrate through the ECM/tissue and detach themselves from it to reach the blood circulation to find new organ sites to target. Metastatic cells express mesenchymal markers such as α-SMA, fibroblast specific protein-1 (FSP1; also known as S100A4), vimentin (or desmin) [22]. These EMT markers are particularly expressed in the cells localised in the invasion front of the tumour [3,23,24], as demonstrated in models in ovarian, colon, oesophageal and bronchial cancers [23,25,26,27,28].

We have also reported that in smokers, though especially marked in COPD subjects, EMT is active in airway epithelial basal cells [29,30]. Further, EMT in large airways is characteristic of Type-III EMT on the basis of Rbm hyper-vascularity which is another characteristic feature, thus forming a microenvironment for the development of lung cancer. In contrast, a process more resembling Type-II EMT is predominant in small airways, thus more pro-fibrotic and perhaps likely to be more related to small airway fibrosis, obstruction and obliteration [30].

## 3. Potential Factors Driving EMT and Its Signalling Pathways

The transition of epithelial cells into mesenchymal cells, in development or pathologically, follows a common and conserved programme with specific hallmarks. However, it also has an inherent flexibility and some variation depending on the context, e.g., cell type, organ/tissue and the signals that activate the EMT programme; partial expression is common. Indeed the EMT subtypes are themselves dependent on such physiological context [16], which differentially alter basal cell gene expression and activation signatures which define the mesenchymal phenotype. Variations occur in degree of increased cell protrusions, due to rearrangements of the intracellular microtubule network, with switching of cytokeratin-rich to vimentin-rich networks during the EMT process. This leads to increased motility [31], and in many cases the ability via matrix metalloprotein production to degrade extracellular matrix (ECM) proteins which is inherent in invasive behaviour. Additionally, cells that have undergone EMT acquire variable resistance to senescence and apoptosis. Thus, the presence of EMT activity is recognised through the presence of such dynamic and diverse properties of activated mesenchymal cells with active in-process transition of epithelial cells with some residual epitopes.

**Figure 1:**
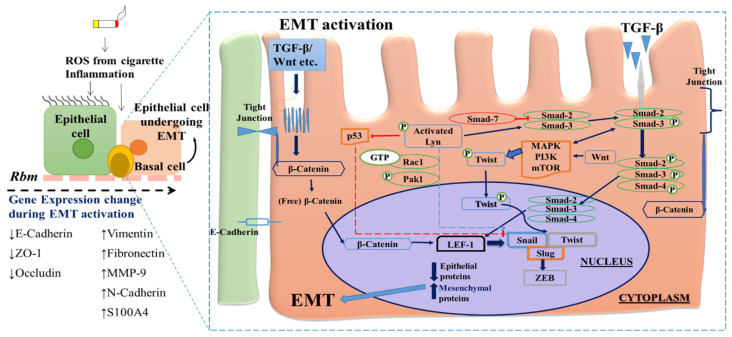
Epithelial-Mesenchymal Transition (EMT)-associated pathways/transcriptional factors as potential therapeutic targets. External stimulus (such as cigarette smoke, pollutants, and luminal infection/inflammation) “activate” the airway epithelium resulting in the increased production of transforming growth factor-β (TGF-β) and other EMT drivers (e.g., Wnt). The sequence is generation of basal (stem) cell signalling molecules (e.g., β-catenin, SMADs and kinases), then elevation of activity of gene transcription factors (e.g., Snail, Twist, Slug etc.), and finally production of EMT-related mesenchymal proteins and suppression of normal epithelial markers.

A limited number of signalling pathways seems to play key roles in EMT regulation. These include those driven by transforming growth factor (TGF), bone morphogenic protein (BMP), fibroblast growth factor (FGF), epidermal growth factor receptor (EGFR), hepatocyte growth factor (HGF), their frequently tyrosine-kinase trans-cell membrane receptors, and TGFβ/SMAD, Wnt/β-catenin, and Notch pathways [32,33] (Figure 1). However, there is considerable redundancy with common shared downstream mechanism and modulating kinases that could be strategically targeted therapeutically.

Indeed, the cancer regulatory protein p53 and its upstream regulatory kinases also seem to be involved in EMT activation. p53 is negatively regulated by the oxidative stress-activated non-receptor tyrosine Src-family kinase Lyn, which has ubiquitous inflammatory and pro-malignant roles in many organ system [34]; in epithelial cells its upregulation and activation through phosphorylation enhances EMT activity [35]. The pathway seems to be that Lyn activation inhibits p53 by which its direct inhibition of EMT activation is removed, i.e., the brake on the other upstream activators which are also switched on by oxidant stress is removed. In human airway epithelial cells from smokers there was a direct correlation found between Lyn and EMT expression [36]. As well as p53 inhibition, this relationship of Lyn to EMT in airway epithelial cells may be due to increasing phosphorylation of Smad EMT-transcription factors in the classic TGFβ1 EMT-inducing pathway, either at the Smad level itself, or through TGFβ1-level activation of this growth factor or its tyrosine kinase receptor system [36]. Other strategic points where effects of TGFβ1 and Wnt stimulation converge, and also where conventional inflammatory pathways cross with EMT activation, are the PI3K/ALF/mTOR and TGFβ-activated kinase (TAK1 or Map3k7) pathways [37,38]. These are highly complex sets of mutually cross-enhancing mechanisms which provide ample targets for future drug therapies.

(Pre-)malignant cells undergoing EMT exhibit both morphological changes and molecular alterations, as observed by decreased epithelial markers expression, including E-cadherin, ZO-1, and occludin, and increased expression of mesenchymal markers, including, S100A4, vimentin, N-cadherin and fibronectin [29,30,39,40]. Boutet et al. have also reported up-regulation of several EMT-associated transcription factors in metastatic cells, including Snail, Twist, Zeb, and others. TGF-β plays a large role in activating Snail, which in turn down-regulates cadherin-16 and HNF-1β which seems to enhance the move to EMT [41].

In addition to the specific EMT-signature transcription factor programs (e.g., SMAD, Snail, Zeb, Twist, etc.), particular miRNAs with either pro- and anti-EMT activity (such as miR-200, miR-205 and miR-21) and also epigenetic regulators have become evident in regulation of EMT-inducing cell plasticity and phenotypic alteration during carcinogenesis [42]. As well as promoting the transformation of early stage primary tumours into invasive malignancies, EMT has also been implicated in the very generation of such early stage cancer cells with stem cell-like characteristics. These subtypes of cells not only exhibit increased tumour-initiating capabilities but also potential for self-renewal (tumour recurrence), and EMT-related resistance to apoptosis and to chemotherapeutic agents [43,44,45]. Such EMT involvement in primary carcinogenesis as well as in accelerated tumour spread and metastasis subsequently has been studied in epithelial cancer stem cells (CSC) in breast [46], pancreatic [47], colorectal [48] and prostate cancer [49], although data are currently still limited for NSCLC. This issue of a primary cancerogenic role for EMT in lung cancer urgently needs further clarification.

## 4. Identification of EMT

Identification of EMT requires a selection of specific immune-markers, but also detailed review of classic phenotypic changes in the state of the tissue. In COPD for instance, we were alerted to the possibility of active airway EMT by observing an irregular and fragmented epithelial reticular basement membrane which was hyper-cellular and hyper-vascular [30]. This was confirmed by loss of the epithelial phenotype in basal cells as characterised by decrease in the expression of epithelial proteins including junction proteins (E-cadherin and zonula occludens-1), cytokeratins of the cytoskeleton and a decreased expression of the surface protein MUC1 (Mucin 1, cell surface associated). Although the loss of these proteins has been widely described during EMT, the acquisition of mesenchymal markers is slower and more difficult to prove. However, markers available for defining the mesenchymal phenotype are vimentin, α-SMA, fibroblast specific protein (FSP1 or S100A4), desmin, fibronectin and the production of MMPs [50]. Nonetheless, vimentin is not expressed by all fibroblasts and is also present in leukocytes and endothelial cells [51]. α-SMA is expressed by myofibroblasts that normally represent only a fraction of the activated fibroblasts, but these increase markedly in the presence of EMT [52,53,54]. Finally, FSP1 (S100A4) is expressed by inflammatory cells, as well as endothelial cells and smooth muscle cells [55,56,57,58]. This non-specificity of mesenchymal markers highlights the difficulty in confirming the presence of EMT, and the need to use multiple markers to characterise the process, and also if possible to double stain for both epithelial markers and a mesenchymal marker in the same transitioning cell. Thus, in COPD we found such co-expression in cells in the basal epithelial area and also in clefts in the reticular basement membrane cells [29].

## 5. EMT and Tumour Metastasis

The importance of the EMT program in tumour development and progression has been established in the past two decades with a rapidly growing number of studies demonstrating its activation during the process of malignant progression [44,59]. We have already made the point that more work is needed in lung cancer on whether EMT is involved in the primary epithelial cancer development [33].

In the epithelial carcinomas, in which the contributions of the EMT program to cancer cell phenotypes have been most intensively studied, EMT-induced mesenchymal traits seems to enable carcinoma cells to complete many of the steps in the invasion-metastasis cascade, including the local invasion by neoplastic cells at the primary tumour site, intravasation into blood vessels, translocation through the circulation, extravasation into the parenchyma of distant tissues, and survival as micrometastatic deposits [3,60]. As noted above in the context of non-malignant cells, it is also rare for carcinoma cells to lose all epithelial traits and gain a full spectrum of mesenchymal characteristics. An important, though rare, variant, reflecting the same principle of heterogeneity, is provided by carcino-sarcomas, in which distinct epithelial and mesenchymal compartments coexist in the tumour although derived from a common cellular precursor [61].

More than 90% of cancer-associated deaths are associated with metastatic disease rather than by the localized primary tumours [60,62]. The increased motility/invasiveness associated with the mesenchymal cell has linked this tumour metastasis with expression of the malignant cell’s EMT program, in which individual cell separation from the primary tumour mass can be considered as the first step of the invasion-metastasis cascade [60]. In addition, a majority of studies using both mouse models and human cancer cell-line culture have demonstrated that induction of an EMT program allows carcinoma cells to lose cell-cell junctions, degrade local basement membrane via elevated expression of various matrix-degrading enzymes, e.g., MMPs, and access to regional blood vessels, as evidence of migration and invasion as single cells [63,64,65,66]. Further histopathological analysis has revealed that at the tumour invasive front, which is in close proximity to the desmoplastic stroma, a higher proportion of cells express an EMT phenotype with loss of epithelial markers and cell-cell junctions and increased expression of mesenchymal markers [23,39,67,68].

Further, EMT-mediated neo-angiogenesis has been proposed as a mechanism of enhanced dissemination of tumour cells into the systemic circulation [69]. However, the notion that cells with EMT phenotypes preferentially disseminate into the circulation is contradicted by the findings that both epithelial and mesenchymal cells are found in the peripheral blood [70]. Thus, other modes of cell migration, including passive shedding [71], collective and clustered migration [72], early dissemination at the stage of carcinoma in situ [73], and migration into vascularized areas inside the tumour mass, have been suggested as contributing to tumour dissemination. These are not mutually exclusive mechanisms, however, and movement of individual cells between phenotypic epithelial and mesenchymal appearances may well occur at different stages of the individual cells’s journey.

## 6. EMT-MET Interaction

Thus, as well as EMT, its reversal process of mesenchymal-to-epithelial transition (MET) has been suggested to play important roles in metastatic dissemination of carcinomas, so that in different phases during the metastatic process carcinoma cells can attain either a fully epithelial or a fully mesenchymal state [3]. This allows tumour cells that have a motile EMT phenotype to allow intravasation of blood capillaries at the primary tumour site and also to extravasate into the distant organ, in general must revert back to a largely epithelial phenotype in order to grow optimally in the secondary site and become a clinically relevant and detectable mass. Indeed, it has been suggested that there is mutual cooperation between epithelial and mesenchymal cell phenotypes such that the mesenchymal form just “paves the way” [74]. Although scarce, there is evidence supporting the notion that CTCs could be used as an early diagnostic tool to monitor lung cancer initiation in patients with COPD [75]. Unfortunately, the study was limited by small number of participants, i.e., CTCs were detected in only 3% (5 out of 168) of COPD patients. Nonetheless, a small number of CTCs exhibited expression of vimentin with a weak associated cytokeratin expression. This indicates that monitoring CTCs in ‘at risk’ COPD patients could be useful in early detection of lung cancer, however, larger studies are needed to test and confirm these findings.

It is likely that it is fluctuating activity of EMT-TFs and microRNA families that regulate EMT which causes this full or sometimes partial switching between EMT and MET cancer cell phenotype phases [76]. However, detailed discussion of the literature on this is beyond the scope of this article. Further, MET-independent metastasis do occur but the mechanisms involved in these cases remain elusive. More information on the control mechanism involved here could be important in stimulating new therapeutic strategies.

## 7. Lung Cancer

Histologically, lung cancer is characterised into two main types: small cell carcinoma and non-small cell carcinoma (the latter including especially adeno and squamous cell carcinoma). Pathological association of smoking with the development of COPD and then lung cancer in that group, is of high clinical significance. This association may well have links with EMT and its downstream pathways. We have observed that the EMT in large airways in smokers is ubiquitous, and characteristic of so-called Type-III EMT with angiogenesis in the reticular basement membrane and epithelium, while Type-II EMT is predominant in small airways, i.e., without new and abnormal blood vessels. As mentioned earlier, this is consistent with the major smoking-related pathologies in the two compartments: in large airways the development of lung cancer, while in small airway obstruction and obliteration (perhaps as well as cancer) [30]. We have failed to find differences in EMT activity between squamous cell- and adeno-carcinomas [39], nor in the relationship between EMT activity in the tumours versus the large and small airways from which they are likely to have emerged [39]. Whether Type-II EMT in the small airways is also in fact pro-cancer development is another area where further work is needed for clarification.

Shi and colleagues were the first to report that in lung cancer cells, poor patient outcomes were correlated with elevated EMT markers [77]. Interestingly, fibroblasts associated with lung cancer (CAF) demonstrated microarray gene expression of proteins regulated by TGF-β [78]. Thus, TGF-β is a master regulator of cancer development operative at a number of levels and not only directly at stimulating EMT [79]. Sustained TGF-β signalling (for example by autocrine signalling to drive sustained zinc finger protein ZEB expression and resultant reduced levels and activity of miR200, through its DNA methylation) may be required for the maintenance of EMT and also for metastasis in several mouse lung cancer models [80,81,82]. At the same time, lung cancer somehow avoids the autocrine growth inhibitory effect of TGF-β_2_ in most NSCLC and SCLC, and restoration of this anomaly might be a potential target for therapeutic intervention [83,84].

Another EMT driver, Wnt signalling, has been demonstrated in many cancers and has recently emerged as a critical pathway in lung carcinogenesis. Cells expressing Wnt-1 are resistant to therapies that mediate apoptosis. Wnt-1 and Wnt-2 have been found to be over-expressed in NSCLC lines and other primary tumour tissues. In addition, an anti-Wnt-1 monoclonal antibody has been shown to suppress tumour growth [85].

We know that both SCLC and NSCLC require an active Hh signalling pathway, yet another route into EMT. Furthermore, it has been shown that an antagonist of the Hh pathway inhibits proliferation of NSCLC cell lines with an Hh autocrine loop. However, Hh signalling in lung cancer may also feed into control of cell proliferation, and provison of a viable blood supply via VEGF and platelet-derived growth factor (PDGF) signalling pathways [86].

Notch signalling, also frequently associated with EMT gene-programme activation, is also required for lung cancer development, as illustrated by studies on knockout mice. Notch receptors and ligand are both elevated in NSCLC lines. Blockade of Notch pathway activation, using gamma secretase inhibition, shows increased apoptosis and reduced in vivo and in vitro NSCLC tumour growth [87]. However, in contrast, forced activation of Notch signalling in tumour-associated macrophages enhanced their anti-tumour capacity [88].

A recent study by Marzieh Rezaei etal highlighted an association of Human Papillomavirus (HPV) infection with EMT-associated lung cancer development. They demonstrated high expression of EMT-promoting HPV genes (E-2, E6 and E7), high TGF-β and N-cadherin expression in HPV + ve lung cancer patients. Similarly, Slug gene overexpression along with resultant E-cadherin gene down-regulation was marked in HPV-associated lung cancer. These EMT molecular signatures were strongly correlated with the stage of lung cancer, being more in stage IV and lowest in stage IA and IB [89].

We have already dealt with the interaction of Lyn kinase activation and inhibition of the anti-epithelial cancer protein p53, with then knock-on effects on EMT by taking off this anti-EMT brake. Further, and as discussed, Lyn is also likely to interact with the TGF-β/Smad pathway. Not much is yet known about the relative importance of these and other complex kinase systems and whether they have independent effects on cancer development or whether this is all or at least mainly mediated through EMT activation.

Finally for this section, a novel human finding [39] was that EGFR and expression of EMT-related proteins were markedly high at the peripheral leading edge of NSCLCs, both adeno and squamous-cancers and related to tumour and clinical characteristics associated with poor prognosis. There is a potential here for developing prognostic markers. The relationships between degree of EMT-related tumour bio-marker expression and those in the corresponding airway epithelium and reticular basement membrane, suggest that the airway microenvironment from which the tumour arises influence the cancer’s subsequent natural history.

## 8. COPD and Lung Cancer

There has been a quite a number of individual studies over the past two decades showing now convincingly that COPD itself is a strong risk factor for lung cancer, and importantly, to be independent of the smoking that caused the COPD, although of course this and other common background factors can be challenging to untangle [90,91]. The consensus quantitative increase in risk for lung cancer with COPD is 5-fold; meaning that up to 70% of lung cancers could be related to COPD. It is very possible that this is an underestimate and indeed the majority of COPD worldwide may be undiagnosed [92]. In addition, the disease has its predominant physiological effect in the small airways but the conventional way of detecting COPD with spirometry underestimates this quite appreciably [93]. Of course, very many smokers never get to have lung function in the first place [94]. Analysis of genetic influences that bring together COPD and lung cancer risk is beyond the scope of this paper, but we recently reviewed this mutuality in the context of more broadly covering COPD pathogenesis [33].

We have already alluded to our human tissue findings of airway epithelial EMT activity in smokers. Invariably this is even greater in subjects with COPD [33]. Furthermore, we have teased out the roles of TGβ/Smad and β-catenin-Snail1-Twist pathways, and the strong relationship between EMT activity and degree of obstructive lung function abnormality [33,39].

## 9. Drugs That Affect Both COPD and Lung Cancer and Their Interrelationship

Even more intriguing is the growing evidence for a protective effect of inhaled corticosteroid (ICS) against lung cancer risk in COPD. Ge et al. [95] recently systematically reviewed nine studies incorporating almost 182,000 COPD patients, representing 1.1 million person-years. This showed a relatively uniform reduction in cancer risk of 30% with ICS use. However, this is likely an underestimate of the effect, as shown by Raymakers et al. [96] the actual protective effect is closer to 70% if the first 2 years of ICS use are excluded from such analyses to take into account cancers already developed.

Statins have long been thought of as potentially useful in COPD, both in terms of morbidity and mortality, but have not been taken up for this indication [61]. As for ICS, there is also strong population pharmaco-epidemiological evidence that statin use (aimed at cholesterol reduction) coincidentally reduces the risk of lung cancer in COPD, especially for NSCLC. This effect is again large, with about a 40–60% reduction in cancer development if again a 2 year use threshold is applied and if medication is used for more than 6 months [97,98]; this effect varies with dose and lipophilicity of specific drugs [97,98]. Further, survival in lung cancer patients is significantly better, by up to 20% if they are co-incidentally taking statins post-operatively [99].

Although an airway luminal anti-innate inflammatory effect has been the favoured theory for these cancer-protective effects of ICS and statins, there is little evidence for this, although an anti-inflammatory effect may contribute to reducing oxidative stress and thus activation of the epithelium, although the exact mechanisms by which anti-inflammatories impart this effect is not yet known in the context of COPD-lung cancer. However, in in vitro cancer cell cultures both drugs have an inhibitory effect on malignancy status through influencing the usually mutually inhibitory balance between the anti-cancer protein p53 and pro-malignant epithelial basal cell EMT activity [34,100,101,102]. There is a sting in this tail, in that with particular mutations in the p53 gene the interaction between p53 and EMT may be positive and the effect of statins acceleratory [102].

In vivo human data is difficult to obtain, but in a small proof-of-concept RCT of high dose ICS using airway biopsy in COPD patients, Sohal et al. showed an inhibitory effect on EMT markers in airway epithelium [103]. In the same study analysis of EMT-related blood vessels, part of the pro-malignant airway environment, showed reversion towards normal with ICS [104].

## 10. Potential EMT-Targeted Therapies

It is our contention, based on the balance of evidence as we see it, that EMT plays a major (and possibly fundamental) pathogenic role in COPD airway remodelling disease, AND related lung cancer development. However, there may be a number of pathways activated by oxidant stress on the airway epithelium, for which EMT may be the final common pathway into airway remodelling and obstruction/obliteration as well as tumorigenesis. Because of the upstream complexity, it might be that drugs directly inhibiting the EMT process should be the first to be trialled for prevention of COPD/lung cancer in vulnerable people.

Fortunately, EMT is now increasingly being considered as treatable and thus being targeted in various stages of drug development (Table 1). Notably, multiple EMT-related drugs have been emerging over the last decade in the oncology literature [105,106,107,108,109], with seven more in PubMed just in the last year, with one directed specifically at NSCLC [110]. Meanwhile in IPF, anti-fibrotic drugs (such as nintedanib and pirfenidone) which seem to work through inhibition of TGF-β pathways [111] have been introduced with effect. All of these drug modalities hold great promise for translation to inhibition of COPD development and through that prophylaxis against lung cancer.

However, the drugs already used for short-term objectives in COPD may themselves be strategically repurposed to focus on their own long-term anti-EMT preventive actions; these might be directed at EMT pathways themselves and/or through damping innate luminal inflammation and its contribution to airway oxidant burden. As noted earlier, it is notable that inhaled corticosteroids (ICS) have been suggested as being both protective in lung cancer and specifically inhibitory of EMT in human airways [90,98]. Long-acting β-agonists (LABAs), long-acting muscarinic antagonists (LAMAs) and phosphodiesterase (PDE)-inhibitors may also contribute to a direct anti-EMT effect [40], as might urokinase-type plasminogen activator receptor (uPAR) inhibition [112].

As we have discussed, statins also decrease lung cancer risk while being shown to reduce EMT activity and through this cancer aggressiveness [105,106,107,108,109]. Designer small molecules which inhibit the epigenetic reader BRD4 have been shown to switch off EMT activity and airway remodelling via a TGF-β related mechanism [113]. The same could be said for the anti-EMT and anti-cancer effects of the multi-biological mTOR modulator Resveratrol [114]. miRNAs are also being directed at this mTOR target [38]. It has also been shown that humble aspirin has potent anti-EMT action in NSCLC cells by decreasing mTOR expression via p300 acting on its promotor region [115]. Interestingly, another more specific COX2 cyclooxygenase inhibitor, celecoxib, inhibits EMT in epithelial tumour cells through miRNA-145 down-regulation of the TGFβ-receptor and the Smad pathway [116].

Indeed, the possibilities for anti-EMT therapeutic interventions in long-term preventive anti-lung cancer clinical studies are so wide that much thought will be needed about where to start; perhaps ICS (or even better triple once a day ICS/LABA/LAMA combination) plus a lipophilic statin would be a good place, given their widespread use and safety record already in COPD patients.

## 11. Conclusions

We would suggest that the very concept of COPD, and its therapeutic culture, needs major reconsideration and reworking. The diagnosis of COPD at a particular physiological cut-off is arbitrary and ignores the major recent insight that smoking-related airway disease starts years before FEV_1_ declines or significant symptoms start and take the patient to their doctor. Yet, current management guidelines and practice essentially focus almost exclusively on symptoms and acute exacerbations which are really manifestations of advanced disease. As discussed above, inter-disciplinary research suggests strong common links between EMT, COPD and lung cancer, and the mechanisms involved are beginning to be well understood. However, these concepts are still not fully integrated into the mainstream.

We would suggest that there should be a corresponding paradigm change for drug treatment of COPD. This should target primary preventive therapeutic strategies, with mediation(s) introduced prophylactically at a much earlier stage in middle age, and to address basic pathogenic mechanism of both airway destruction/obstruction and the concurrent lung cancer risk. Those selected for trials of this approach in the first instance would be those who can be shown with spirometric monitoring to have a more rapid “pre-COPD” downhill trajectory of lung function airflow indices [117].

Thus, it may well be that our current focus on short-term symptomatic outcomes, with a pathogenic emphasis on airway luminal cellular innate inflammatory changes rather than long-term airway remodelling and related cancerogenesis, has led to us to overlook the potential for repurposing commonly used COPD/asthma drugs and with a different timescale and a new preventive strategic purpose. As well as a potential for repurposing existing drugs, our suggested re-evaluation of the COPD paradigm also suggests trialling of novel anti-EMT drugs already in the pipeline.

## Figures and Tables

**Table 1:** Some of the Molecules designed for Targeting EMT in Lung Cancer.

Agents	Potential Targets	Potential Effects	Indication	Drug Developmental Stage
Bufalin	TGF-β pathway	EMT inhibition	NSCLC	Preclinical
SU11274	C-Met	Overcomes resistance to EGFR-targeted therapies	NSCLC	Preclinical
RO4929097	Notch signalling	Inhibits EMT markers and cell migration or invasion	NSCLC	Phase II
R428 (BGB324)	Axl	Overcomes resistance to EGFR-targeted therapies	NSCLC	Phase I/II
LY3039478	Notch signalling	Inhibits EMT markers and cell migration/invasion	Advanced solid tumours	Phase I
CX-4945	CK2	Inhibits PI3K-Akt pathway and TGF-β1-induced EMT	Advanced solid tumours	Phase I
SGI-7079	VEGFR. Axl	Inhibits Axl and overcomes resistance to EGFR-targeted therapies	NSCLC	Preclinical
Moscatilin	Snail, Slug, and vimentin	Inhibits EMT and sensitizes anoikis	NSCLC	In vitro (cell line)

## Data Availability

Not applicable.

## Abbreviations

α-SMA	α smooth muscle actin
COPD	chronic obstructive pulmonary disease
CSC	cancer stem cells
CTCs	circulating tumour cells
ECM	extracellular matrix
EMT	epithelial-to-mesenchymal transition
ICS	inhaled corticosteroids
LABAs	Long-acting β-agonists
LAMA	long-acting muscarinic antagonists
MET	mesenchymal-to-epithelial transition
miRNA	micro-RNA
MMP	matrix metalloproteinases
NSCLC	Non-small cell lung cancer
TGF	transforming growth factor
ZO-1	zonula occuludens-1
